# Intestinal Helminth Infections and Their Association With Nutritional Status and Diarrheal Diseases Among School‐Aged Children in North Gondar Zone, Ethiopia

**DOI:** 10.1155/japr/1613179

**Published:** 2026-06-30

**Authors:** Gebre Ayanaw Alula, Tilahun Endale, Maru Wudu

**Affiliations:** ^1^ Department of Biology, College of Natural and Computational Science, Debark University, Debark, Ethiopia, dku.edu.et

**Keywords:** anemia, diarrhea, Ethiopia, intestinal helminth infection, school-aged children, stunting

## Abstract

Intestinal helminth infections remain a major public health problem in low‐ and middle‐income countries, particularly among school‐aged children, where they contribute to malnutrition and diarrheal diseases. This study is aimed at assessing the prevalence of intestinal helminth infections and their association with nutritional status and diarrheal diseases among school‐aged children in the North Gondar Zone, Ethiopia. An institution‐based cross‐sectional study was conducted from September to December 2025 among 374 children selected using a multistage sampling technique. Data were collected using structured questionnaires, anthropometric measurements, and hemoglobin assessment, and stool examination was performed using direct wet mount and formol‐ether concentration techniques. Multivariable logistic regression analysis was used to identify factors associated with helminth infection and health outcomes, and statistical significance was declared at p < 0.05. The prevalence of intestinal helminth infection was 14.97% (*n* = 56). Helminth infection was associated with higher odds of stunting (AOR = 1.82; 95% CI: 1.05–3.12), anemia (AOR = 2.05; 95% CI: 1.10–3.82), and diarrhea (AOR = 2.76; 95% CI: 1.52–5.01). Furthermore, poor sanitation, unsafe drinking water, inadequate hygiene practices, and low dietary diversity were significant predictors of helminth infection and were also associated with increased risk of stunting, anemia, and diarrheal disease. Overall, intestinal helminth infections and modifiable environmental and nutritional factors were independently associated with adverse child health outcomes. These findings highlight the importance of integrated interventions combining deworming programs with improvements in water, sanitation, and hygiene (WASH), along with nutrition‐sensitive strategies, to improve child health in the study area.

## 1. Introduction

Intestinal helminth infections remain one of the most common neglected public health challenges worldwide, particularly in low‐ and middle‐income countries where poverty, inadequate sanitation, and limited access to safe water sustain transmission [1]. According to global estimates, over 1 billion people are infected with intestinal helminths, including *Ascaris lumbricoides*, *Trichuris trichiura*, and hookworms, with school‐aged children carrying the highest burden [2]. The highest prevalence is observed in sub‐Saharan Africa, Southeast Asia, and parts of Latin America, where environmental conditions and socioeconomic constraints favor ongoing transmission and reinfection [3, 4]. In Ethiopia, studies consistently report moderate to high prevalence of intestinal helminth infections, particularly in rural and semiurban communities, despite ongoing control and deworming programs [3, 4].

From an epidemiological perspective, the distribution and persistence of helminth infections are closely linked to environmental, behavioral, and sociodemographic factors [5]. Poor sanitation, lack of access to clean water, open defecation, and inadequate hygiene practices significantly increase the risk of exposure [6]. Children living in rural areas, those from low socioeconomic backgrounds, and those with limited health access are particularly vulnerable, creating a clear pattern of inequality in infection risk and disease burden [7].

Helminth infections are known to affect nutritional status through multiple biological mechanisms. These parasites impair nutrient absorption, induce chronic intestinal inflammation, and, in the case of hookworm infections, cause ongoing blood loss that leads to iron deficiency and anemia [8, 9]. As a result, infected children are at increased risk of stunting, wasting, underweight, and micronutrient deficiencies [10]. Over time, these effects compromise physical growth, immune function, and cognitive development, ultimately affecting educational attainment and long‐term productivity [11]. This makes helminth infections not only a parasitic disease but also a major contributor to the global burden of malnutrition [12].

In addition, helminth infections contribute to diarrheal diseases by damaging the intestinal mucosa and altering host immune responses, thereby increasing susceptibility to enteric pathogens [13]. Diarrhea further exacerbates malnutrition through reduced food intake, impaired nutrient absorption, and increased fluid and nutrient loss, creating a vicious cycle between infection and poor nutritional outcomes [14]. This interconnected relationship highlights the critical role of environmental factors, particularly water, sanitation, and hygiene (WASH), in maintaining the transmission cycle [15].

Despite national deworming efforts in Ethiopia, intestinal helminth infections remain a persistent public health concern, especially in regions such as the North Gondar Zone [16]. However, there is limited context‐specific evidence that simultaneously examines the relationship between helminth infections, nutritional status, and diarrheal diseases while considering environmental, behavioral, and sociodemographic determinants. Most existing studies focus primarily on prevalence estimates or isolated health outcomes, with limited assessment of the cumulative and interacting effects of helminth infection, poor WASH conditions, and nutritional factors on child health [17, 18].

Although several Ethiopian studies have reported the prevalence of intestinal helminths, few studies have simultaneously examined the interconnected relationship between helminth infection, nutritional status, diarrheal disease, and WASH‐related determinants using integrated multivariable approaches among school‐aged children in Northwest Ethiopia. Moreover, evidence on the combined burden and interaction of environmental and parasitic risk factors remains limited.

Understanding these interconnected and cumulative relationships is important because adverse child health outcomes in low‐resource settings are often driven by multiple overlapping biological and environmental factors rather than by single exposures alone [19]. Assessing the combined contribution of helminth infection and WASH‐related conditions may therefore provide more comprehensive epidemiological evidence to support integrated intervention strategies.

Therefore, generating localized and comprehensive evidence is essential to better understand the combined effects of helminth infections on child health. This study was conducted to assess the association between intestinal helminth infections and nutritional outcomes (stunting, wasting, and anemia), as well as diarrheal diseases among school‐aged children in the North Gondar Zone, Ethiopia, while accounting for key environmental and behavioral factors. Unlike many previous studies that examined isolated predictors, the present study additionally evaluated the combined burden of intestinal helminth infection and WASH‐related factors on child health outcomes to better understand their cumulative and potentially synergistic effects. By adopting an integrated approach, the study is aimed at providing evidence that can inform more effective and holistic public health interventions to break the cycle of infection and malnutrition.

## 2. Materials and Methods

### 2.1. Study Area and Period

This study was carried out in the North Gondar Zone, located in the Amhara Regional State in Northwest Ethiopia, about 830 km from Addis Ababa and 103 km from Gondar. The zone lies between 13°8 ^′^ N latitude and 37°54 ^′^ E longitude and features a wide range of landscapes, including both highland and lowland areas, with elevations reaching up to about 2850 m above sea level. These geographic and environmental variations contribute to differences in climate, sanitation, and living conditions across the area.

The zone is home to a predominantly rural and semiurban population of over 900,000 people, where access to clean water, improved sanitation, and healthcare services is not uniform. Rainfall patterns, temperature variations (ranging from 12°C to 20°C), and everyday hygiene and sanitation practices all play an important role in shaping the transmission of intestinal helminth infections in the community.

Because of this combination of diverse environmental conditions and varying access to basic services, North Gondar Zone provides a suitable setting for studying intestinal helminth infections and their health impacts among school‐aged children. The study was conducted over 4 months, from September to December 2025.

### 2.2. Study Design

An institution‐based cross‐sectional study design was employed to assess the prevalence of intestinal helminth infections and their association with nutritional status and diarrheal diseases among school‐aged children in the North Gondar Zone, Ethiopia.

### 2.3. Source and Study Population

The source population comprised all school‐aged children enrolled in primary schools in the North Gondar Zone. The study population consisted of selected students from the sampled schools who met the eligibility criteria during the study period. School‐aged children were targeted because they are among the most vulnerable groups to intestinal helminth infections and related outcomes, including malnutrition and diarrheal diseases.

### 2.4. Inclusion and Exclusion Criteria

School‐aged children who were enrolled in the selected primary schools and who provided assent with parental/guardian consent were included in the study.

Children who were critically ill or who had received antihelminthic treatment within 2 weeks prior to data collection were excluded from the study. This 2‐week exclusion window was applied to minimize the potential influence of recent drug administration on stool examination results, as antihelminthic treatment can temporarily reduce or eliminate detectable parasite eggs in fecal samples, leading to false‐negative findings. This timeframe is consistent with field‐based parasitological surveys, where short posttreatment exclusion periods are commonly applied to improve diagnostic accuracy while maintaining operational feasibility [20, 21].

### 2.5. Sample Size Determination

The sample size for this study was determined using the single population proportion formula, *n* = *Z* ^2^p(1 − p)d^2^, where *Z* = 1.96 for a 95% confidence level, *p* = 0.127 (based on a previous study conducted in Ethiopia by Teshale et al. [22], and *d* = 0.05 margin of error. The initial sample size was calculated to be 170. After applying a design effect of 2 to account for the multistage sampling technique, the sample size became 340. Adding a 10% nonresponse rate, the final sample size was 374 participants.

### 2.6. Sampling Technique

A multistage sampling technique was employed to select study participants. First, all schools in the North Gondar Zone were identified using the official registry obtained from the Zonal Education Office. From this list, schools were selected using a simple random sampling method to ensure representativeness and reduce selection bias. Second, after school selection, proportional allocation was applied to determine the number of study participants from each school based on the total number of enrolled school‐aged children, ensuring that schools with larger student populations contributed proportionally more participants to the study sample. Finally, within each selected school, systematic random sampling was used to select individual participants. The sampling frame consisted of the official student registration lists obtained from each school. The sampling interval (k) was calculated using the formula *k* = *N*/*n*, where *N* represents the total number of eligible students in each school and *n* represents the allocated sample size. A random starting point between 1 and *k* was selected, and thereafter every kth student on the list was included until the required sample size was achieved.

### 2.7. Data Collection Procedures

Data were collected using structured questionnaires, anthropometric measurements, hemoglobin assessment, and laboratory parasitological examination. All procedures were conducted following standardized protocols to ensure data quality and reliability.

#### 2.7.1. Questionnaire Survey

A structured questionnaire was used to collect data on sociodemographic characteristics, environmental conditions, behavioral practices, sanitation and hygiene practices, and history of diarrheal illness. The questionnaire was developed based on relevant literature and adapted from standardized and previously validated tools, including World Health Organization (WHO) child health questionnaires [23], Demographic and Health Survey (DHS)/UNICEF survey instruments [24], and findings from similar Ethiopian WASH‐related studies [25]. The tool was initially prepared in English, translated into the local language, and back‐translated to ensure consistency and semantic equivalence. To ensure validity, relevance, and clarity, the questionnaire was pretested on a sample outside the study area, and necessary modifications were incorporated before the actual data collection.

#### 2.7.2. Anthropometric Measurements

Anthropometric measurements were conducted following WHO standard procedures. Body weight was measured using a calibrated digital weighing scale (SECA model or equivalent) to the nearest 0.1 kg, and height was measured using a portable stadiometer to the nearest 0.1 cm. The equipment was calibrated daily before data collection to ensure measurement accuracy and reliability.

Nutritional status was assessed using height‐for‐age (HAZ), weight‐for‐height (WHZ), and weight‐for‐age (WAZ) Z‐scores calculated using WHO AnthroPlus software. However, WAZ was not used as a primary index for nutritional assessment among older school‐aged children because WHO AnthroPlus does not recommend weight‐for‐age for children above 10 years due to its limited interpretability in this age group. Therefore, stunting and wasting were defined based on HAZ and WHZ, respectively, with Z‐scores below −2 standard deviations (SD) from the median classified as malnutrition indicators.

#### 2.7.3. Hemoglobin Assessment

Hemoglobin concentration was measured using a portable HemoCue hemoglobin analyzer. Capillary blood samples were obtained via finger prick using sterile, single‐use lancets after disinfecting the puncture site with 70% alcohol. The first drop of blood was wiped away using sterile gauze, and the second drop was used for analysis according to the manufacturer′s instructions. Hemoglobin measurement was performed immediately at the field site to avoid sample degradation. Anemia was classified based on WHO age‐specific hemoglobin cutoff values.

#### 2.7.4. Stool Sample Collection and Laboratory Examination

Fresh stool samples were collected in clean, labeled, leak‐proof containers and transported to the laboratory within 1–2 h of collection. Laboratory analysis was performed using both direct wet mount and formol‐ether concentration techniques following standard parasitological procedures as described in WHO laboratory manuals and established diagnostic protocols [26, 27]. Samples were examined microscopically by trained laboratory personnel for detection and identification of helminth eggs and larvae. Quality control procedures were implemented throughout the laboratory process to ensure accuracy and reliability of results.

### 2.8. Data Quality Assurance

Data quality was ensured through several measures. The questionnaire was pretested in a similar population outside the study area to ensure clarity and consistency. Data collectors and supervisors were trained on data collection procedures, anthropometric measurements, and laboratory techniques. Daily supervision and regular checks were conducted to identify and correct errors. Laboratory analyses followed standardized protocols, and equipment was regularly calibrated to ensure accuracy and reliability of measurements.

### 2.9. Data Analysis

Data were entered into SPSS Version 30 and carefully checked for completeness, consistency, and accuracy before analysis. Descriptive statistics, including frequencies, percentages, means, and SD, were used to summarize the data.

Bivariate logistic regression analysis was performed to identify candidate variables associated with intestinal helminth infection, nutritional outcomes (stunting, wasting, and anemia), and diarrheal diseases. Variables with a *p* value less than 0.20 in the bivariate analysis were included in the multivariable logistic regression model to control for potential confounding factors. Subsequently, multivariable logistic regression analysis was conducted to identify independent predictors of the outcome variables.

Adjusted odds ratios (AOR) with 95% confidence intervals (CI) were computed to measure the strength of associations, and statistical significance was declared at a *p* value less than 0.05.

In addition to individual outcome analyses, a combined burden analysis was conducted to assess the cumulative effects of intestinal helminth infection and WASH‐related factors on child health outcomes. Composite exposure categories were created by combining helminth infection status with key WASH variables, including poor hygiene practices, unsafe drinking water sources, and lack of latrine utilization. Multivariable logistic regression models were then applied to assess the combined effect of these exposures on stunting, anemia, and diarrheal diseases.

Furthermore, interaction terms between intestinal helminth infection and selected WASH‐related variables were included in the regression models to assess potential effect modification and synergistic relationships between parasitic infection and environmental risk factors on adverse health outcomes.

Model fitness was assessed using the Hosmer–Lemeshow goodness‐of‐fit test, with *p* > 0.05 indicating an acceptable model fit. Multicollinearity among independent variables was evaluated using the variance inflation factor (VIF), and variables with VIF values less than 10 were considered to have no significant multicollinearity.

## 3. Results

### 3.1. Sociodemographic Characteristics of Study Participants

A total of 374 school‐aged children participated in the study. The mean age of the participants was 11.0 ± 2.4 years. Males accounted for 58.3% (*n* = 218), whereas females represented 41.7% (*n* = 156).

Regarding age distribution, 24.9% of participants were aged 6–9 years, 44.9% were 10–12 years, and 30.2% were 13–15 years. In terms of residence, the majority of the children (63.6%) were from rural areas, whereas 36.4% were from urban settings.

Regarding educational level, 34.5% of participants were in Grades 1–3, 40.9% were in Grades 4–6, and 24.6% were in Grades 7–8. Concerning parental educational status, 41.2% of parents had no formal education, 36.4% had primary education, and 22.5% had attained secondary education or higher (Table 1).

**Table 1:** Sociodemographic characteristics of study participants (*n* = 374).

Variable	Category	Number examined (*n*)	Percentage (%)
Sex	Male	218	58.3
	Female	156	41.7

Age group (years)	6–9	93	24.9
	10–12	168	44.9
	13–15	113	30.2

Residence	Urban	136	36.4
	Rural	238	63.6

Grade level	Grade 1–3	129	34.5
	Grade 4–6	153	40.9
	Grade 7–8	92	24.6

Parental education	No formal	154	41.2
	Primary	136	36.4
	Secondary and above	84	22.5

### 3.2. Prevalence of Intestinal Helminth Infections

The overall prevalence of intestinal helminth infections among the study participants was 14.97% (*n* = 56). *A. lumbricoides* was the most commonly identified parasite, with a prevalence of 7.22% (*n* = 27), followed by hookworm (3.74%, *n* = 14) and *Taenia* spp. (1.87%, *n* = 7). Other detected parasites included *Strongyloides stercoralis* (1.34%, *n* = 5) and *Schistosoma mansoni* (0.80%, *n* = 3).

Single infections were more common than mixed infections, with polyparasitism observed in 1.34% (*n* = 5) of participants (Table 2).

**Table 2:** Prevalence of intestinal helminth infections.

Parasite species	Infected (*n*)	Prevalence (%)
*Ascaris lumbricoides*	27	7.22
Hookworm	14	3.74
*Strongyloides stercoralis*	5	1.34
*Taenia* spp.	7	1.87
*Schistosoma mansoni*	3	0.8
Mixed infections	5	1.34
Total	56	14.97

#### 3.2.1. Factors Associated With Intestinal Helminth Infections

The prevalence of intestinal helminth infections was stratified and analyzed according to sociodemographic characteristics, including age, sex, and residence (Table 3). A chi‐square test was used to assess differences in prevalence across categories.

**Table 3:** Sociodemographic factors associated with intestinal helminth infections among school‐aged children.

Variable	Category	Total (*n*)	Infected (*n*)	Prevalence (%)	*χ* ^2^
Age group	6–9 years	93	18	19.4	
	10–12 years	168	22	13.1	
	13–15 years	113	16	14.2	6.84

Sex	Male	218	37	16.9	
	Female	156	19	12.2	4.12

Residence	Urban	136	15	11.1	
	Rural	238	41	17.2	5.76

Infection prevalence varied by age group (*χ* ^2^ = 6.84, *p* = 0.033). The highest prevalence was observed among children aged 6–9 years (19.4%), followed by those aged 13–15 years (14.2%) and 10–12 years (13.1%).

Higher prevalence was observed among male children (16.9%) compared with females (12.2%) (*χ* ^2^ = 4.12, *p* = 0.042). Regarding residence, rural children had a higher prevalence (17.2%) than urban children (11.1%) (*χ* ^2^ = 5.76, *p* = 0.016) (Table 3).

#### 3.2.2. Association Between Specific Helminth Species and Diarrheal Disease

Species‐specific analysis was conducted to describe the distribution of diarrheal disease among children infected with different intestinal helminth species (Table 4).

**Table 4:** Association between specific helminth species and diarrheal disease.

Helminth species	Infected (*n*)	Diarrhea *n* (%)	No diarrhea *n* (%)
*Ascaris lumbricoides*	27	14 (51.9)	13 (48.1)
Hookworm	14	7 (50.0)	7 (50.0)
*Taenia* spp.	7	2 (28.6)	5 (71.4)
*Strongyloides stercoralis*	5	2 (40.0)	3 (60.0)
*Schistosoma mansoni*	3	1 (33.3)	2 (66.7)
Mixed infection	5	3 (60.0)	2 (40.0)
Total	56	29 (51.8)	27 (48.2)

The proportion of diarrheal disease varied across helminth species. Diarrhea was observed in 51.9% of children infected with *A. lumbricoides* and 50.0% of those infected with hookworm. The proportion of diarrhea was 40.0% among children infected with *S. stercoralis*, 33.3% among those infected with *S. mansoni*, and 28.6% among those infected with *Taenia* spp. Children with mixed infections had a diarrhea proportion of 60.0%. No species‐specific odds ratios were calculated due to small subgroup sizes (Table 4).

### 3.3. Nutritional Status of Study Participants

The nutritional status of 374 school‐aged children was assessed using WHO growth indicators, including stunting (height‐for‐age), wasting (weight‐for‐height), and anemia based on hemoglobin concentration. The prevalence of stunting was 29.5% (*n* = 110), wasting was 13.8% (*n* = 52), and anemia was 14.2% (*n* = 53) (Table 5).

**Table 5:** Nutritional status of participants.

Indicator	Number examined (*n*)	Prevalence (%)
Stunting	110	29.5
Wasting	52	13.8
Anemia	53	14.2

Stunting, wasting, and anemia were reported across all age groups. The highest proportion of all three indicators was observed among children aged 6–9 years, followed by those aged 10–12 years and 13–15 years. Across sex categories, slightly higher proportions of stunting, wasting, and anemia were observed among male children compared with females. These patterns are presented descriptively in Table 5 and in the relevant sociodemographic distribution tables (Tables 1 and 3).

### 3.4. Prevalence of Diarrheal Diseases

The prevalence of diarrheal diseases among the study participants was 21.7% (*n* = 81). Among those, 68.0% (*n* = 55) reported a single episode of diarrhea, whereas 32.0% (*n* = 26) experienced recurrent episodes (Table 6).

**Table 6:** Prevalence of diarrheal diseases.

Condition	Number examined (*n*)	Percentage (%)
Diarrhea (yes)	81	21.7
Diarrhea (no)	293	78.3

### 3.5. Association Between Helminth Infection and Nutritional Status

The prevalence of malnutrition among the study participants was high, with stunting being the most common form (29.5%), followed by anemia (14.2%) and wasting (13.8%).

In the bivariate analysis, intestinal helminth infection was significantly associated with stunting, anemia, and wasting (Table 7). However, after adjustment for potential confounders, significant associations were observed only for stunting and anemia.

**Table 7:** Association between intestinal helminth infection and nutritional outcomes among school‐aged children (*n* = 374).

Outcome	Helminth infected *n* (%)	Not infected *n* (%)	COR (95% CI)	*p*	AOR (95% CI)	*p*
Stunting	28 (50.0%)	82 (25.8%)	2.15 (1.30–3.55)	0.003	1.82 (1.05–3.12)	0.031
Wasting	11 (19.6%)	41 (12.9%)	1.78 (1.01–3.12)	0.046	1.54 (0.82–2.88)	0.176
Anemia	17 (30.4%)	36 (11.3%)	2.42 (1.38–4.24)	0.002	2.05 (1.10–3.82)	0.024

Children infected with intestinal helminths had higher odds of being stunted (AOR = 1.82; 95% CI: 1.05–3.12; *p* = 0.031) and anemic (AOR = 2.05; 95% CI: 1.10–3.82; *p* = 0.024) compared with their noninfected counterparts.

Although infected children also showed increased odds of wasting, this association was not statistically significant in the multivariable model (AOR = 1.54; 95% CI: 0.82–2.88; *p* = 0.176) (Table 7).

### 3.6. Factors Associated With Intestinal Helminth Infections

A range of sociodemographic, behavioral, and environmental factors was examined for their association with intestinal helminth infections (Table 8).

**Table 8:** Factors associated with helminth infections.

Risk factor	Category	Number examined (*n*)	Prevalence (*n*/%)	COR (95% CI)	*p*	AOR (95% CI)	*p*
Age	6–9 years	93	18.3%	1	—	1	—
	10–12 years	168	15.5%	0.82 (0.48–1.41)	0.471	0.78 (0.44–1.37)	0.386
	13–15 years	113	12.4%	0.64 (0.36–1.12)	0.118	0.59 (0.32–1.08)	0.087
Sex	Female	156	11.5%	1	—	1	—
	Male	218	17.4%	1.62 (0.97–2.70)	0.066	1.45 (0.84–2.51)	0.182
Poor hand hygiene	Good hygiene	202	8.9%	1	—	1	—
	Poor hygiene	172	22.1%	3.26 (1.89–5.61)	< 0.001	2.74 (1.52–4.93)	0.001
Lack of latrine use	Uses latrine	226	7.5%	1	—	1	—
	Does not use latrine	148	24.3%	3.89 (2.21–6.83)	< 0.001	3.11 (1.70–5.69)	< 0.001
Unsafe water source	Safe water	179	8.4%	1	—	1	—
	Unsafe water	195	21.5%	2.94 (1.71–5.05)	< 0.001	2.36 (1.30–4.27)	0.005
Not wearing shoes	Wearing shoes	211	7.6%	1	—	1	—
	Not wearing shoes	163	20.9%	2.78 (1.62–4.77)	< 0.001	2.21 (1.22–4.02)	0.009
Low dietary diversity	High dietary diversity	186	8.1%	1	—	1	—
	Low dietary diversity	188	19.7%	1.61 (1.40–3.16)	0.039	1.13 (0.85–1.98)	0.064
Raw/unwashed food	Washed/cooked food	232	6.9%	1	—	1	—
	Raw/unwashed food	142	23.2%	2.12 (1.78–4.46)	0.027	1.58 (0.81–2.72)	0.051

In the multivariable analysis, age and sex were not significantly associated with helminth infection. Children aged 10–12 years (AOR = 0.78; 95% CI: 0.44–1.37; *p* = 0.386) and 13–15 years (AOR = 0.59; 95% CI: 0.32–1.08; *p* = 0.087) had lower odds of infection compared with children aged 6–9 years, although these differences were not statistically significant. Male children had higher odds of infection than females, but the association was not significant (AOR = 1.45; 95% CI: 0.84–2.51; *p* = 0.182).

In contrast, behavioral and environmental factors were significantly associated with intestinal helminth infections. Poor hand hygiene was associated with increased odds of infection (AOR = 2.74; 95% CI: 1.52–4.93; *p* = 0.001). Lack of latrine use showed the strongest association, with more than threefold higher odds of infection (AOR = 3.11; 95% CI: 1.70–5.69; *p* < 0.001).

Children using unsafe water sources (AOR = 2.36; 95% CI: 1.30–4.27; *p* = 0.005) and those not wearing shoes (AOR = 2.21; 95% CI: 1.22–4.02; *p* = 0.009) also had significantly higher odds of infection.

Although low dietary diversity (AOR = 1.13; *p* = 0.064) and consumption of raw or unwashed foods (AOR = 1.58; *p* = 0.051) showed increased odds of infection, these associations were not statistically significant. Overall, intestinal helminth infections were mainly associated with modifiable behavioral and environmental factors rather than sociodemographic characteristics (Table 8).

### 3.7. Association Between Helminth Infection and Diarrhea

The prevalence of diarrhea was higher among children infected with intestinal helminths (48.2%) compared with those without infection (17.3%).

In the bivariate analysis, helminth infection was significantly associated with diarrhea (COR = 3.21; 95% CI: 1.85–5.57; *p* < 0.001). This association remained significant after adjusting for potential confounders, with infected children having nearly three times higher odds of diarrhea (AOR = 2.76; 95% CI: 1.52–5.01; *p* = 0.001) (Table 9).

**Table 9:** Association between helminth infection and diarrheal diseases.

Variable	Total (*n*)	Diarrhea *n* (%)	COR (95% CI)	*p*	AOR (95% CI)	*p*
Helminth infection (yes)	56	27 (48.2)	3.21 (1.85–5.57)	< 0.001	2.76 (1.52–5.01)	0.001
Helminth infection (no)	318	55 (17.3)	1	—	1	—

### 3.8. Combined Effects in Multivariable Model

The multivariable analysis demonstrated that intestinal helminth infection, along with key environmental, behavioral, and nutritional factors, was independently associated with adverse child health outcomes, including stunting, anemia, and diarrhea (Tables 10, 11, and 12).

**Table 10:** Multivariable logistic regression analysis of factors associated with stunting.

Predictor	AOR	95% CI	*p*
Helminth infection	1.82	1.05–3.12	0.031
Poor hygiene practices	1.76	1.10–2.81	0.018
Inadequate sanitation (no latrine use)	2.21	1.34–3.65	0.002
Unsafe water source	1.69	1.05–2.72	0.031
Not wearing shoes	1.58	0.98–2.55	0.061
Low dietary diversity	2.34	1.40–3.90	0.001

**Table 11:** Multivariable logistic regression analysis of factors associated with anemia.

Predictor	AOR	95% CI	*p*
Helminth infection	2.05	1.10–3.82	0.024
Poor hygiene practices	1.24	0.91–1.66	0.076
Inadequate sanitation (no latrine use)	1.92	1.15–3.21	0.013
Unsafe water source	1.71	0.74–1.92	0.054
Not wearing shoes	1.45	0.89–2.37	0.137
Low dietary diversity	2.12	1.28–3.52	0.003

**Table 12:** Multivariable logistic regression analysis of factors associated with diarrhea.

Predictor	AOR	95% CI	*p*
Helminth infection	2.76	1.52–5.01	0.001
Poor hygiene practices	2.48	1.55–3.96	< 0.001
Inadequate sanitation (no latrine use)	2.87	1.70–4.86	< 0.001
Unsafe water source	2.41	1.45–4.00	0.001
Not wearing shoes	1.32	0.93–1.87	0.062
Low dietary diversity	1.51	0.78–2.64	0.058

Children infected with intestinal helminths had higher odds of stunting (AOR = 1.82; 95% CI: 1.05–3.12; *p* = 0.031), anemia (AOR = 2.05; 95% CI: 1.10–3.82; *p* = 0.024), and diarrhea (AOR = 2.76; 95% CI: 1.52–5.01; *p* = 0.001) compared with uninfected children.

WASH‐related factors, including poor hygiene and lack of latrine use, were consistently associated with increased odds of all three outcomes. Unsafe water sources were also significantly associated with stunting and diarrhea, whereas not wearing shoes showed no statistically significant associations with most outcomes.

Nutritional factors played an important role, as low dietary diversity was significantly associated with stunting and anemia, but not with diarrhea, indicating a stronger relationship with nutritional outcomes than infectious morbidity.

Overall, these findings highlight that intestinal helminth infection, together with WASH and nutritional factors, independently contribute to adverse child health outcomes.

### 3.9. Combined Burden and Interaction Effects of Intestinal Helminth Infection and WASH‐Related Factors on Child Health Outcomes

The combined burden analysis showed that the prevalence of adverse health outcomes varied across exposure categories of intestinal helminth infection and WASH conditions.

The prevalence of stunting was 18.6% among children with neither helminth infection nor poor WASH conditions, compared with 24.5% among those with helminth infection only, 33.8% among those exposed only to poor WASH conditions, and 51.2% among children exposed to both helminth infection and poor WASH conditions.

Similarly, the prevalence of anemia increased from 9.4% in the unexposed group to 18.2% (helminth infection only), 21.4% (poor WASH only), and 38.6% (combined exposure). Diarrheal disease followed a similar pattern, increasing from 11.2% in the unexposed group to 29.6%, 35.7%, and 57.9% across the same exposure categories (Table 13).

**Table 13:** Combined effects of intestinal helminth infection and poor WASH conditions on child health outcomes.

Exposure category	Stunting *n* (%)	Anemia *n* (%)	Diarrhea *n* (%)	AOR (95% CI) for stunting	*p*
No helminth infection + good WASH conditions	18.6	9.4	11.2	1	—
Helminth infection only	24.5	18.2	29.6	1.72 (1.01–2.95)	0.041
Poor WASH conditions only	33.8	21.4	35.7	2.11 (1.29–3.44)	0.003
Helminth infection + poor WASH conditions	51.2	38.6	57.9	4.28 (2.11–8.66)	< 0.001

In multivariable logistic regression analysis, children exposed to both helminth infection and poor WASH conditions had higher odds of stunting compared with unexposed children (AOR = 4.28; 95% CI: 2.11–8.66; *p* < 0.001). Helminth infection only (AOR = 1.72; 95% CI: 1.01–2.95; *p* = 0.041) and poor WASH conditions only (AOR = 2.11; 95% CI: 1.29–3.44; *p* = 0.003) were also associated with increased odds of stunting.

Interaction terms between helminth infection and selected WASH‐related variables were tested. Statistically significant interaction effects were observed between helminth infection and hygiene practices for stunting, between helminth infection and unsafe water source for diarrhea, and between helminth infection and dietary diversity for anemia and stunting (Table 13).

### 3.10. Multiple Outcome Burden Analysis

The multiple outcome burden analysis examined the distribution of single, double, and triple health burdens (stunting, anemia, and diarrheal disease) among children by helminth infection status.

Single burden was observed in 54.1% of noninfected children and 39.3% of infected children. Double burden was more frequent among infected children (41.1%) compared with noninfected children (28.9%). Similarly, triple burden was observed in 14.3% of infected children compared with 4.4% of noninfected children (Table 14).

**Table 14:** Multiple outcome burden among school‐aged children by helminth infection status.

Exposure group	Single burden *n* (%)	Double burden *n* (%)	Triple burden *n* (%)
No helminth infection (*n* = 318)	172 (54.1%)	92 (28.9%)	14 (4.4%)
Helminth infection (*n* = 56)	22 (39.3%)	23 (41.1%)	8 (14.3%)
Total (*n* = 374)	194 (51.9%)	115 (30.7%)	22 (5.9%)

## 4. Discussion

This study demonstrated that intestinal helminth infections remain a significant public health concern among school‐aged children in the North Gondar Zone, with an overall prevalence of 14.97%. This finding is consistent with reports from other parts of Ethiopia, where prevalence typically ranges between 12% and 30% [27–29]. It is also lower than estimates from some high‐burden settings in sub‐Saharan Africa and global estimates exceeding 37% in low‐ and middle‐income countries [30]. These differences may reflect variations in sanitation coverage, environmental exposure, diagnostic techniques, and ongoing deworming interventions [31].

The predominance of *A. lumbricoides* followed by hookworm is consistent with patterns reported across Ethiopia and other tropical regions [32, 33]. *A. lumbricoides* is widely recognized as the most prevalent soil‐transmitted helminth globally, whereas hookworm thrives in warm, moist environments that support larval survival and transmission [34]. The species distribution observed in this study reflects continued environmental contamination and inadequate sanitation, which sustain transmission cycles in endemic communities [35]. Importantly, species‐specific differences may partly explain variation in clinical outcomes: *A. lumbricoides* is more strongly associated with intestinal malabsorption and diarrhea, whereas hookworm infection is primarily linked to chronic intestinal blood loss and iron deficiency anemia.

In this study, intestinal helminth infection was significantly associated with stunting and anemia, but not wasting after adjustment for confounders. These findings are consistent with evidence from Ethiopia and other endemic settings, as well as global meta‐analyses showing that helminth infections are strongly associated with chronic undernutrition and anemia in children [36, 37]. The underlying mechanisms linking helminth infection to these outcomes include reduced nutrient absorption due to intestinal mucosal damage, chronic inflammatory responses, and iron loss in the case of hookworm infection, which directly contributes to iron deficiency anemia [38]. The lack of a significant association with wasting suggests that acute malnutrition is more likely influenced by short‐term factors such as recent illness episodes or food insecurity rather than chronic parasitic infection.

A key contribution of this study is the significant association between helminth infection and diarrheal disease. Children infected with intestinal helminths had nearly three times higher odds of diarrhea compared with noninfected children. This finding is consistent with studies from other low‐resource settings [39, 40]. Helminth infections may predispose children to diarrhea through disruption of intestinal mucosal integrity, altered gut immunity, and increased susceptibility to secondary bacterial and protozoal infections [13]. This interaction may also create a reinforcing cycle in which diarrhea further worsens nutritional deficits through reduced food intake and impaired nutrient absorption [41].

Environmental and behavioral factors, particularly WASH‐related determinants, were significantly associated with helminth infection and all major health outcomes in this study. Specifically, lack of latrine use, unsafe drinking water, and poor hygiene practices were independently associated with increased odds of infection, stunting, anemia, and diarrhea [42]. These findings are consistent with studies conducted in Ethiopia and other endemic regions, where open defecation and unsafe water sources remain major drivers of transmission [43, 44]. Importantly, these associations are based on the current study′s multivariable analysis, indicating that they are independent predictors rather than external evidence.

Dietary diversity was also significantly associated with stunting and anemia. Children with low dietary diversity had higher odds of these outcomes, consistent with evidence from Ethiopia and other LMICs [45]. Poor dietary diversity contributes to micronutrient deficiencies and weakens immune function, increasing susceptibility to infection and reducing recovery capacity.

Although not statistically significant in the adjusted model, factors such as not wearing shoes showed a positive association with helminth infection. This finding is consistent with previous evidence linking barefoot exposure to increased risk of hookworm infection [46]. However, the stronger effects observed for sanitation and hygiene‐related variables suggest that environmental exposure plays a more dominant role than individual behavior alone in this setting.

These findings highlight the interconnected relationship between intestinal helminth infection, malnutrition, diarrheal disease, and WASH‐related factors among school‐aged children, emphasizing the importance of integrated control strategies.

### 4.1. Limitations of the Study

This study has several limitations that should be considered when interpreting the findings. First, the cross‐sectional study design limits the ability to establish causal relationships between intestinal helminth infections, WASH‐related factors, and health outcomes such as stunting, anemia, and diarrhea. Although significant associations were identified, the temporal sequence and directionality of these relationships cannot be determined.

Second, data on behavioral and environmental factors, including hygiene practices and sanitation behaviors, were based on self‐reports. This may introduce recall bias and social desirability bias, potentially leading to under‐ or over‐reporting of certain practices. In addition, although multivariable analysis was performed to control for confounding, residual confounding may still exist due to unmeasured factors such as detailed dietary intake, micronutrient supplementation, household socioeconomic status, and parental education.

Third, the parasitological diagnostic techniques used (direct wet mount and formol‐ether concentration) may have underestimated the true prevalence of intestinal helminth infections, particularly for low‐intensity infections or intermittent egg excretion. Furthermore, the study did not assess infection intensity or seasonal variation, which could have provided additional insight into transmission dynamics and disease severity.

Finally, as the study was conducted in a specific geographic area within the North Gondar Zone, the findings may have limited generalizability to other settings with different environmental, cultural, and socioeconomic conditions.

## 5. Conclusion and Recommendations

This study demonstrates that intestinal helminth infections remain an important public health problem among school‐aged children in the North Gondar Zone, Ethiopia. Helminth infection was significantly associated with increased odds of stunting, anemia, and diarrheal diseases. In addition, key environmental and behavioral factors, particularly poor sanitation and inadequate hygiene practices, were independently associated with these adverse health outcomes, whereas low dietary diversity was significantly linked to poor nutritional status.

Overall, the findings highlight that intestinal helminth infection, malnutrition, diarrheal disease, and WASH‐related factors are strongly interconnected and collectively contribute to a syndemic of poor child health outcomes in the study area. These relationships should be interpreted in light of the cross‐sectional study design, which limits causal inference.

From a public health perspective, the findings indicate that deworming alone is insufficient to sustainably reduce the burden of intestinal helminth infections and related morbidities. Integrated interventions combining preventive chemotherapy with improvements in WASH, as well as nutrition‐sensitive strategies, are required. Strengthening WASH infrastructure, promoting sustained hygiene behavior change, and improving access to safe drinking water are essential components of a comprehensive control strategy. In addition, interventions aimed at improving dietary diversity should be reinforced to address underlying nutritional deficiencies.

Therefore, public health efforts should prioritize a coordinated multisectoral approach involving health, education, water, and agriculture sectors. School‐based deworming programs should be strengthened and integrated with hygiene education, sanitation promotion, and school feeding or nutrition programs.

Finally, continuous health education and routine monitoring systems should be reinforced at both school and community levels to sustain behavior change, reduce transmission of intestinal helminths, and improve overall child health outcomes in endemic settings.

## Author Contributions

G.A.A. conceived the study, developed the research design, conducted data extraction, performed statistical analysis, and drafted the initial manuscript. T.E. contributed to the study design, supervised the data analysis, and assisted in interpreting the results. M.W. contributed to the methodological framework, participated in data interpretation, and provided critical revisions to the manuscript.

## Funding

No funding was received for this manuscript.

## Disclosure

All authors reviewed, edited, and approved the final version of the manuscript.

## Ethics Statement

Ethical approval for this study was obtained from the Debark University Research Ethics Committee (Ref. No: REC/DKU/011/2025). All methods were performed in accordance with the relevant guidelines and regulations. Written informed consent was obtained from the parents or legal guardians of all participating schoolchildren, as all participants were under 16 years of age. In addition, verbal assent was obtained from the children after explaining the purpose, procedures, potential benefits, and risks of the study in an age‐appropriate manner. Participation was voluntary, and confidentiality of all data was strictly maintained.

## Conflicts of Interest

The authors declare no conflicts of interest.

## Data Availability

The data sets used and/or analyzed during the current study are available from the corresponding author on reasonable request.
